# Characterization of epitope-tagged foot-and-mouth disease virus

**DOI:** 10.1099/vir.0.043521-0

**Published:** 2012-11

**Authors:** Julian Seago, Terry Jackson, Claudia Doel, Elizabeth Fry, David Stuart, Michiel M. Harmsen, Bryan Charleston, Nicholas Juleff

**Affiliations:** 1 Pirbright Laboratory, Institute for Animal Health, Woking, Surrey, GU24 0NF, UK; 2 Division of Structural Biology, Wellcome Trust Centre for Human Genetics, University of Oxford, Roosevelt Drive, Oxford OX3 7BN, UK; 3 Central Veterinary Institute Wageningen UR, Division Virology, PO Box 65, 8200 AB Lelystad, The Netherlands

## Abstract

Foot-and-mouth disease (FMD) is a highly contagious and economically devastating disease of cloven-hoofed animals with an almost-worldwide distribution. Conventional FMD vaccines consisting of chemically inactivated viruses have aided in the eradication of FMD from Europe and remain the main tool for control in endemic countries. Although significant steps have been made to improve the quality of vaccines, such as improved methods of antigen concentration and purification, manufacturing processes are technically demanding and expensive. Consequently, there is large variation in the quality of vaccines distributed in FMD-endemic countries compared with those manufactured for emergency use in FMD-free countries. Here, we have used reverse genetics to introduce haemagglutinin (HA) and FLAG tags into the foot-and-mouth disease virus (FMDV) capsid. HA- and FLAG-tagged FMDVs were infectious, with a plaque morphology similar to the non-tagged parental infectious copy virus and the field virus. The tagged viruses utilized integrin-mediated cell entry and retained the tag epitopes over serial passages. In addition, infectious HA- and FLAG-tagged FMDVs were readily purified from small-scale cultures using commercial antibodies. Tagged FMDV offers a feasible alternative to the current methods of vaccine concentration and purification, a potential to develop FMD vaccine conjugates and a unique tool for FMDV research.

## Introduction

Foot-and-mouth disease (FMD) is a highly contagious and economically important disease of cloven-hoofed animals, affecting domesticated ruminants and pigs, as well as a large number of wildlife species. The causal agent is FMD virus (FMDV), a member of the family *Picornaviridae*, genus *Aphthovirus*. The FMDV particle consists of a non-enveloped icosahedral protein shell (capsid) containing a single-stranded, positive-sense RNA genome approximately 8500 nt in length (Forss *et al.*, 1984). The FMDV genome encodes a single polyprotein that is processed proteolytically to generate a set of intermediate precursors and final mature proteins that are required for virus replication and assembly. The N-terminal P1 region of the polyprotein is processed into four structural proteins [VP1 (1D), VP2 (1B), VP3 (1C), and VP4 (1A)], whilst the P2 and P3 regions are processed to yield the non-structural proteins (NSP) essential for viral RNA replication, as well as for disruption of cellular processes that restrict virus replication.

The structural proteins VP4 and VP2 are initially produced as a precursor (VP0), which assembles along with a single molecule each of VP1 and VP3 to form a protomer. Five protomers assemble to form a pentamer, and 12 pentamers assemble to form a capsid that encloses the genomic RNA (Acharya *et al.*, 1989). On encapsidation of the viral RNA, VP0 is cleaved to form VP2 and VP4. The three-dimensional structures of a number of FMDV serotypes have been determined by X-ray crystallography (Curry *et al.*, 1996; Lea *et al.*, 1994, 1995). VP1, VP2 and VP3 fold into eight-stranded β-barrels and form the capsid, whereas VP4 is located inside the capsid along with the viral RNA. VP1, VP2 and VP3 of FMDV are smaller than their counterparts in other picornaviruses, which results in the virion having a smooth appearance. Consequently, FMDV lacks the distinctive surface features such as canyons and pits that have been described for other picornaviruses (Acharya *et al.*, 1989; Hogle *et al.*, 1985). In contrast, the FMDV capsid can be distinguished by a prominent surface-exposed loop connecting the βG and βH strands of VP1 (known as the GH loop of VP1). This loop is conformationally flexible, mediates cell attachment by binding integrin receptors and is a major viral antigenic site (Acharya *et al.*, 1989). It includes a number of conserved residues including the arginine–glycine–aspartate (RGD) motif, which constitutes the main site for integrin recognition (Logan *et al.*, 1993). This site is highly adapted for binding to αvβ6 integrin, forming stable complexes dependent on two conserved residues at positions RGD +1 and RGD +4 (Dicara *et al.*, 2008; Burman *et al.*, 2006).

Advances in reverse genetics and infectious clone technology have enabled the fusion of viral proteins with epitope tags. Such chimeric viruses have been used to study virus replication cycles (Merz *et al.*, 2011), to develop improved methods of virus isolation and concentration using affinity tag purification protocols (Prentoe & Bukh, 2011; Wegelt *et al.*, 2011) and to generate positive marker vaccines (Beach *et al.*, 2011; Holinka *et al.*, 2009). In this study, we have identified a location within the GH loop of VP1 that tolerates the insertion of exogenous epitope tags, while maintaining the interaction with αvβ6 integrin (Lea *et al.*, 1994, 1995). We show that tagged viruses are genetically stable and can be readily purified. We report the construction, characterization and, for the first time, the purification of infectious FMDV with a tagged capsid protein.

## Results

### Insertion of FLAG and HA epitope tags into the GH loop of VP1

**Fig. 1.**
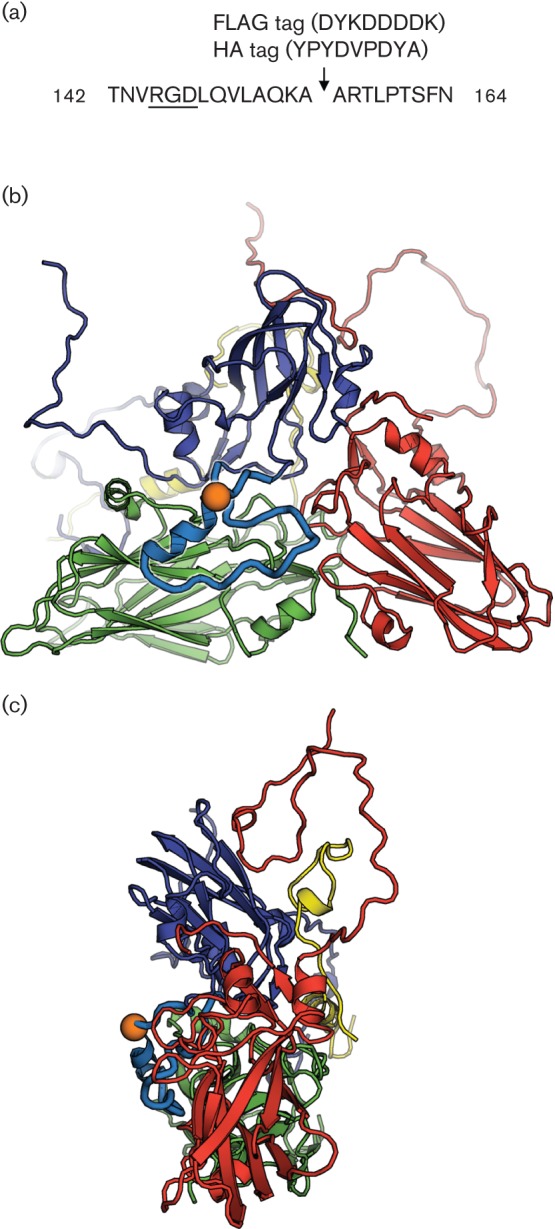
Site of tag insertion in FMDV VP1. (a) The FLAG or HA tag was inserted between alanine 155 and alanine 156 (indicated by an arrow) within the GH loop of VP1. The RGD motif required for integrin-mediated cell entry is underlined. (b, c) Face and side views of an icosahedral protomer of FMDV (O1K strain) drawn in cartoon format. VP1 is depicted in blue, VP2 in green, VP3 in red and VP4 in yellow. The position of the insert, encoding either the FLAG tag or HA tag, in the VP1 GH loop (sky blue) is shown by an orange sphere.

Tagged viruses were derived from infectious copy parental viruses as described in Methods. The GH loop of VP1 was selected as a potential site for epitope tagging. This loop is highly flexible and shows considerable length and sequence variation between different serotypes. Most variation lies downstream of the integrin-attachment site (which comprises the RGD and RGD +1 and RGD +4 residues; residues 145–151). High-affinity binding to integrin αvβ6 also requires the integrity of a helical structure that follows the RGD (Burman *et al.*, 2006; Dicara *et al.*, 2008). Consequently, a site downstream of the integrin-binding site between alanine 155 and alanine 156 within the GH loop of VP1 was selected (Fig. 1). DNA encoding either the HA or the FLAG tag was then inserted into an O1K/O UKG35 FMDV chimera using a PCR-based approach. Sequencing of the respective P1 FMDV stock across the site of insertion confirmed retention of the nucleic acids encoding either the HA or the FLAG epitope.

### Tag expression analysis

**Fig. 2.**
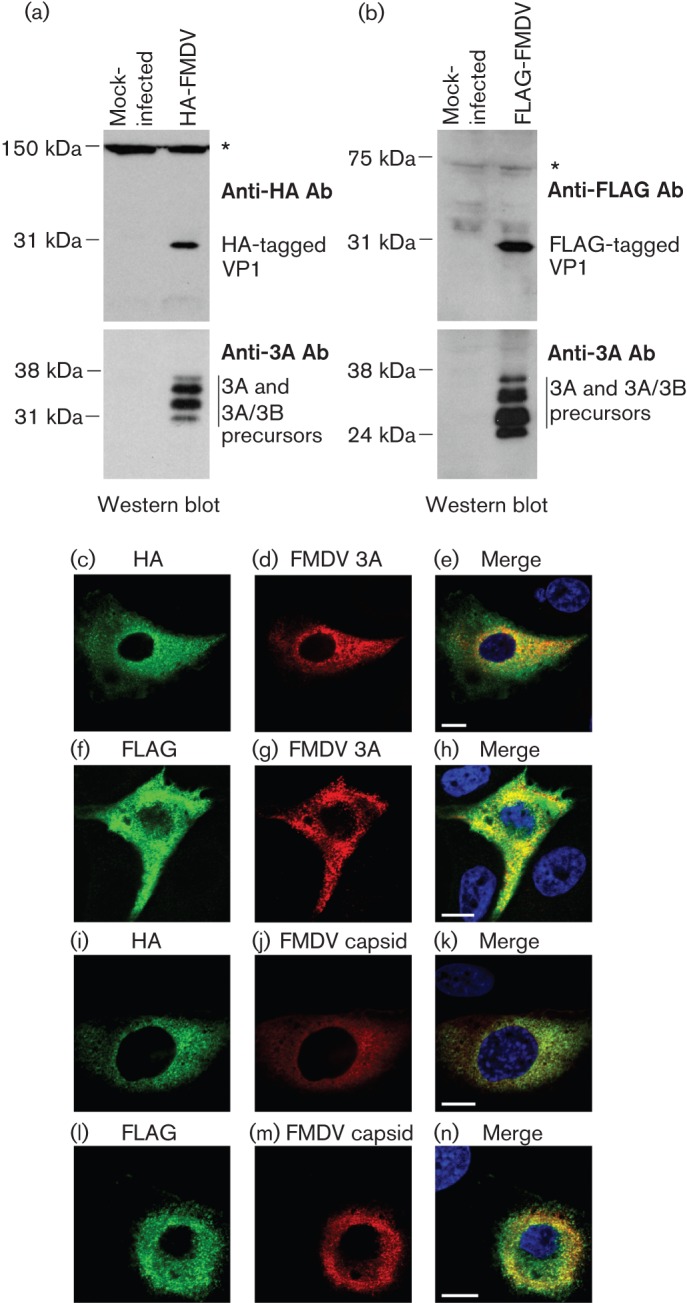
Western blot analysis of whole-cell lysates of goat epithelium cells mock-infected or infected with FMDV O1K/O UKG35 viruses (P1 stock) expressing either the (a) HA tag or (b) FLAG tag in the VP1 capsid protein. The blots were probed for the presence of the respective tag using the HA-7 (anti-HA) and M2 (anti-FLAG) mAbs, and for the non-structural 3A FMDV protein (and 3A/B precursors) using the 2C2 mAb. Asterisks indicate non-specific protein bands and confirm equal loading. (c–n) Goat epithelium cells infected with epitope-tagged FMDV O1K/O UKG35. (c–e) Cells infected with HA-tagged virus; (f–h) cells infected with FLAG-tagged virus. The HA tag and FLAG tag (green) are clearly visible in cells supporting FMDV replication, determined by the presence of the non-structural FMDV protein 3A (red). (i–k) Cells infected with HA-tagged virus; (l–n) cells infected with FLAG-tagged virus. The HA tag and FLAG tag (green) are co-localized with FMDV capsid (red) labelled with mAb BF8, a mAb specific for conformational epitopes on the FMDV capsid (Juleff *et al.*, 2008). Nuclei are stained blue (DAPI). Bars, 10 µm.

Expression of the tagged VP1 capsid protein was confirmed by Western blot analysis of whole-cell lysates prepared from goat epithelium cells infected with P1 FMDV O1K/O UKG35 stock. Fig. 2(a, b) shows that both HA-tagged and FLAG-tagged VP1 were readily detected with molecular masses of the predicted size (about 24 kDa). In addition, to confirm the presence of virus replication, the whole-cell lysates were analysed for the presence of the FMDV non-structural 3A protein and its uncleaved 3A-3B precursors. Expression of the HA or FLAG tag in conjunction with structural and non-structural FMDV proteins was also confirmed by immunofluorescence labelling of cells infected with P1 viral stocks (Fig. 2c–n). As expected, both the HA and FLAG tags co-localized with FMDV capsid.

### Characterization of the tagged viruses

**Fig. 3.**
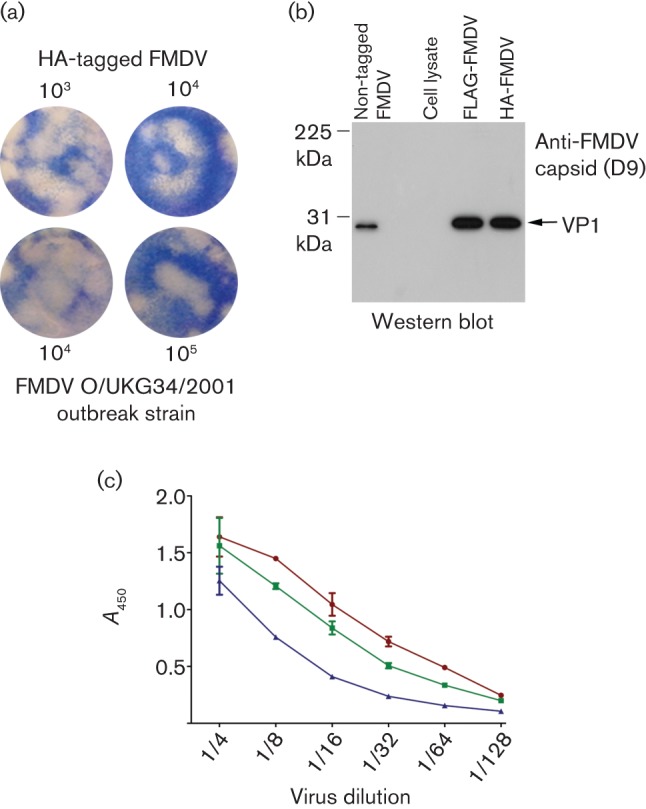
(a) Plaques of similar morphology were observed when confluent monolayers of goat epithelium cells were infected with the indicated dilutions of HA-tagged FMDV O1K/O UKG35 (top) or FMD lesion vesicular fluid from a cow infected with FMDV O/UKG 34/2001 (bottom). Similar results were achieved with FLAG-tagged FMDV O1K/O UKG35 (data not shown). (b) Western blot analysis of tissue-culture supernatants of goat epithelium cells infected with FMDV O1K/O UKG35 not expressing a tag (positive control) or expressing either the HA tag or FLAG tag in the VP1 capsid protein. The blot was probed using a mAb (D9) that recognizes the major antigenic site 1 within the GH loop of O-serotype FMDV. A whole-cell lysate prepared from uninfected goat epithelium cells (negative control) was also analysed. (c) Anti-FMDV sandwich ELISA showing that FMDV O1K/O UKG35 non-tagged (▴), FLAG-tagged (•) and HA-tagged (▪) viruses all interact with mAb D9.

**Fig. 4.**
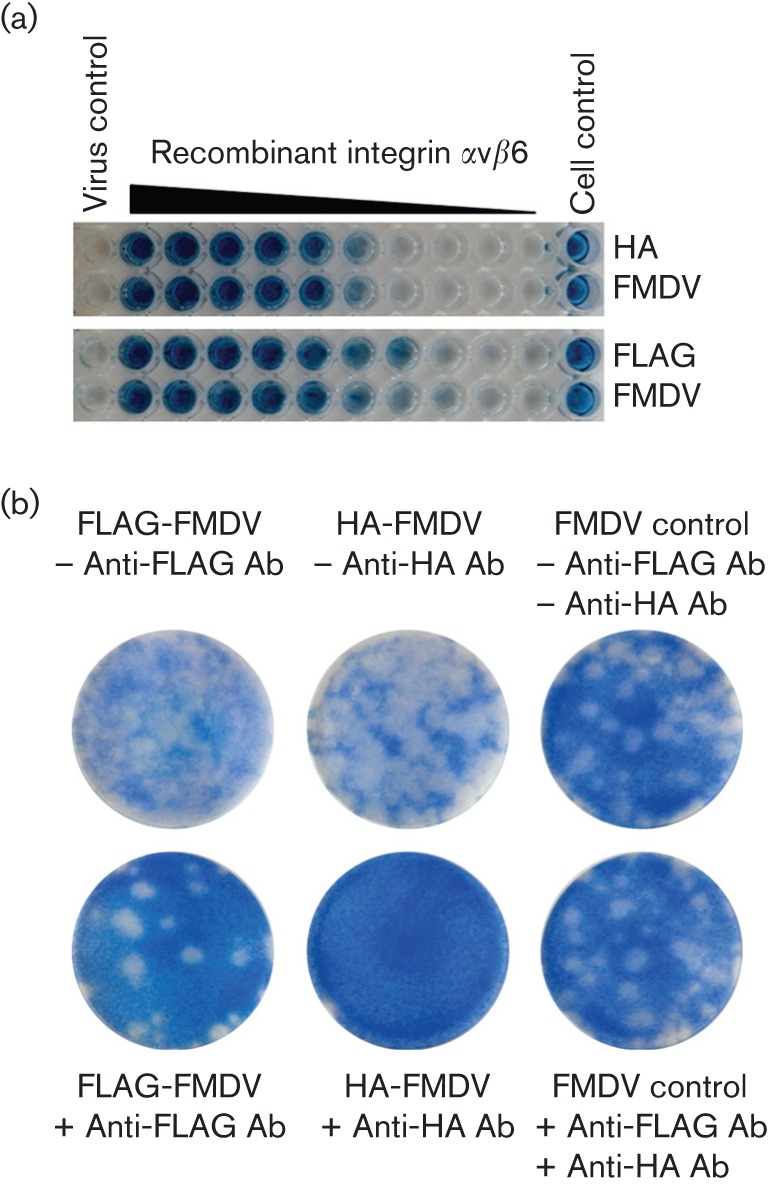
Neutralization assays. (a) HA-tagged and FLAG-tagged FMDV O1K/O UKG35 were incubated with decreasing concentrations of recombinant integrin αvβ6 and then used to infect confluent monolayers of goat epithelium cells. CPE, resulting in no staining, is clearly visible in cells infected with HA-tagged or FLAG-tagged FMDV that had not been incubated with integrin (virus control), whilst no CPE was observed in uninfected cells incubated with the highest concentration of integrin (cell control). (b) FLAG-tagged, HA-tagged or non-tagged parental (FMDV control) FMD O1K/O UKG35 viruses were incubated with the indicated anti-tag antibody and then used to infect confluent monolayers of goat epithelium cells. –, No incubation with antibody (Ab); +, incubation with Ab.

To determine whether the tagged viruses have a similar phenotype to the wild-type virus, plaque assays were performed. HA- and FLAG-tagged FMDV O1K/O UKG35 produced plaques with morphology similar to an FMDV field strain (FMDV O/UKG 34/2001) obtained directly from lesion material (Fig. 3a). Similar plaque morphology was also observed between the parental non-tagged virus and the tagged viruses (see Fig. 4b). Infections of goat epithelium cells with parental non-tagged virus, HA-tagged or FLAG-tagged viruses (at an m.o.i. of 1) all gave total cytopathic effect (CPE) after 5 h, indicating similar replication kinetics (data not shown).

Next, we investigated whether insertion of the tag compromised the antigenic or receptor-binding properties of the virus. Equal quantities of tissue-culture supernatants of goat epithelium cells infected with non-tagged, FLAG-tagged or HA-tagged viruses were analysed by Western blot using a mAb (D9) recognizing the GH loop of VP1 on the FMDV capsid (antigenic site 1) (McCahon *et al.*, 1989). Fig. 3(b) shows that a single band corresponding to VP1 was recognized by D9 in both the non-tagged and tagged FMDV samples. No band was observed in a whole-cell lysate prepared from uninfected goat epithelium cells as a negative control. The recognition of both tagged viruses by antibody D9 was corroborated by ELISA (Fig. 3c), confirming that major antigenic site 1, involved in virus neutralization, is accessible in both HA-tagged and FLAG-tagged viruses.

The ability to infect goat epithelium cells implied the retained use of integrins as receptors. However, due to the proximity of the inserted tags to the RGD motif of the VP1 GH loop, we wanted to confirm that the tagged viruses could interact with integrin αvβ6, the principal receptor utilized by FMDV. To do this, we performed integrin-blocking assays, in which recombinant integrin αvβ6 was pre-incubated with tagged viruses before the infection of goat epithelium cells. Fig. 4(a) shows that pre-incubation with integrin αvβ6 inhibited infection, suggesting that the integrin was able to bind both the HA-tagged and FLAG-tagged viruses. These results confirm that the RGD motif has been maintained in VP1 subunits of the viral capsids and show that the motif is accessible and can interact with integrin αvβ6 in the presence of either the HA tag or FLAG tag expressed in VP1. Next, we investigated whether pre-incubation of the tagged viruses with tag-specific antibodies could also block integrin-mediated infection. Fig. 4(b) clearly shows that HA- or FLAG-specific antibodies effectively neutralize tagged virus and prevent infection of goat epithelium cells. In contrast, no neutralization was observed with parental non-tagged virus in the presence of HA- or FLAG-specific antibodies. Importantly, purification studies (see below) using either the HA- or FLAG-specific antibodies were able to produce infectious viruses, confirming the reversible nature of the observed neutralization.

### Genetic stability studies

**Fig. 5.**
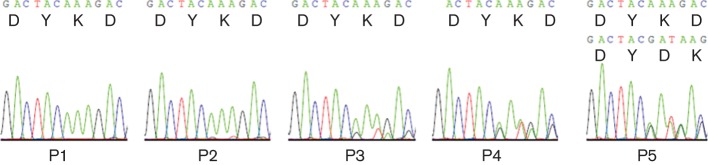
Sequence analysis of FLAG-tagged FMDV O1K/O UKG35 over five consecutive passages (P1–P5) in goat epithelium cells. Only the DYKD residues of the FLAG tag (DYKDDDDK) are shown. In P5, the sequence for both the original peptide-tagged virus and a virus subpopulation with an in-frame deletion of KDDD from the FLAG tag, yielding a DYDK motif, are depicted.

The genetic stability of both HA-tagged and FLAG-tagged O1K/O UKG35 viruses was investigated to determine whether the nucleic acids encoding the epitopes were maintained during five serial passages using goat epithelium cells. For each passage, cells were infected for 24 h, by which time CPE was observed in all cases. Virus stocks (P1, P2, P3, P4 and P5) were then sequenced across the site of insertion in VP1. The HA tag was retained over all five passages (P1–P5) without changes to the nucleic acids encoding the peptide. The FLAG tag was also retained over all five passages; however, analysis of the sequence traces revealed that the P3, P4 and P5 populations consisted of both the original peptide-tagged virus and a virus subpopulation with an alteration to the sequence encoding the FLAG tag (Fig. 5). The alteration comprised an in-frame deletion of a lysine and three consecutive aspartate residues from the FLAG tag (DYKDDDDK), yielding a DYDK motif.

To investigate whether the insertion of either tag resulted in alterations to the structural proteins, the entire capsid-coding regions of both the HA-tagged and FLAG-tagged P5 virus stocks were sequenced. One mutation was observed in the HA-tagged virus, resulting in a threonine to arginine (T to R) amino acid change at position 158 of VP1. No alterations were observed in the capsid of either FLAG-tagged virus population (DYKDDDDK population and DYDK subpopulation). Throughout the multiple passages of both tagged viruses, no changes were identified to the RGD region.

### Purification studies

**Fig. 6.**
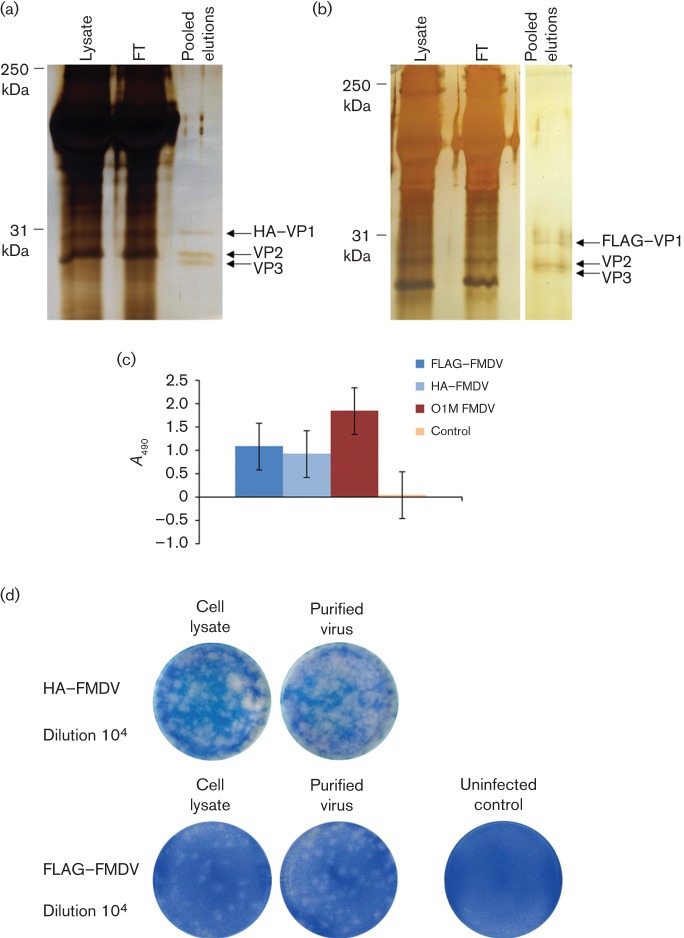
Purification of infectious HA-tagged and FLAG-tagged FMDV O1K/O UKG35. (a, b) Silver-stained gels showing lysate, flow-through (FT) and pooled elution samples of HA-tagged (a) or FLAG-tagged (b) FMDV. Arrows indicate positions of the structural proteins VP1, VP2 and VP3. (c) Purified HA-tagged and FLAG-tagged viruses were analysed by ELISA for intact 146S capsids. Purified non-tagged FMDV (serotype O1M) and a no-virus control served as positive and negative controls, respectively. (d) Plaque assays were performed to compare the infectivity of HA-tagged or FLAG-tagged viruses before purification (cell lysates) and after purification (purified virus). The uninfected control shows a confluent monolayer of goat epithelium cells with no CPE.

Next, purification experiments were performed to determine whether commercially available antibody–agarose bead complexes could be used to capture the HA-tagged and FLAG-tagged viruses from infected cell lysates. Following incubation and wash steps, bound viruses were eluted from the bead complexes using peptide competition for the respective tag. Eluted viruses were visualized by silver staining to assess their purity (Fig. 6a, b). Whole FMDV particles have a sedimentation coefficient of 146S. During FMDV vaccine manufacturing, the golden standard for quantifying the antigen contents is the 146S levels. This method gives information whether the antigen is intact, containing the RNA or not (Van Maanen & Terpstra, 1990). To determine the composition of the eluted viruses, with regard to intact 146S capsids, ELISAs were performed using a llama single-domain antibody fragment that binds specifically to 146S particles (Harmsen *et al.*, 2007). Fig. 6(c) shows that intact 146S particles were present in both the eluted HA-tagged and FLAG-tagged viruses. Sucrose gradient-purified FMDV [O1 Manisa (O1M) serotype (kindly provided by N. Ferris, Institute for Animal Health, Pirbright, UK)], using large-scale conventional sucrose-gradient techniques, served as a positive control. To confirm the infectivity of the eluted viruses, goat epithelium cells were infected and plaque assays were carried out (Fig. 6d).

### Insertion of the HA tag into FMDV O1 Manisa

**Fig. 7.**
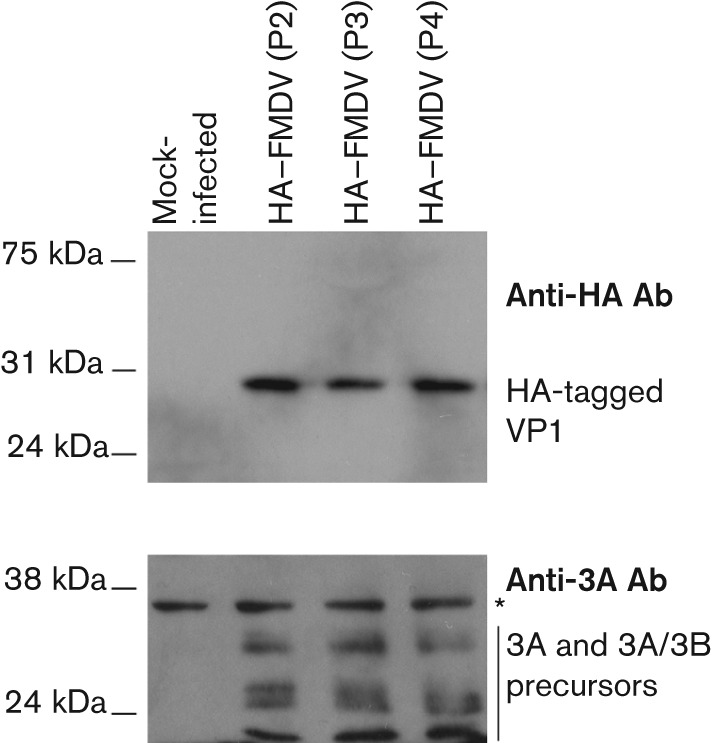
Western blot analysis of whole-cell lysates of goat epithelium cells mock-infected (mock) or infected with FMDV O1 Manisa expressing the HA tag in the VP1 capsid protein. The blot was probed for the presence of the HA tag using a goat anti-HA antibody (QED Bioscience), and for the non-structural 3A FMDV protein (and 3A/B precursors) using the 2C2 mAb. An asterisk indicates a non-specific protein band and confirms equal loading.

To confirm that the insertion site can be used to tag another FMDV strain (serotype O), the HA tag was inserted into the analogous position of a second recombinant infectious copy virus containing the capsid of O1 Manisa in the same genetic background [FMDV O1K (encoding proteins: Lpro, VP4, 2B, 2C, 3A, 3B, 3C, 3D)/O1M (encoding proteins: VP2, VP3, VP1, 2A)]. Western blot analysis of whole-cell extracts prepared from infected goat epithelium cells showed that the HA tag was retained over four consecutive passages (Fig. 7).

## Discussion

Reverse genetics has facilitated the production of viable recombinant viruses with epitope-tagged structural proteins (SP). Examples include the recently reported tagged SP of mammalian reovirus, hepatitis C virus and bovine viral diarrhea virus (Brochu-Lafontaine & Lemay, 2012; Prentoe & Bukh, 2011; Wegelt *et al.*, 2011). Previously, Baranowski *et al.* (2001) produced viable type C FMDV in which residues of the VP1 GH loop were replaced by the FLAG epitope. This loop includes the integrin-binding RGD motif and is a major antigenic site on the capsid that is recognized by neutralizing antibodies. Hence, the resulting tagged virus was unable to interact with integrin receptors or neutralizing antibodies that recognize the VP1 GH loop. More recently, Wang *et al.* (2012) produced recombinant Asia1 FMDVs with insertions in the GH loop. These insertions were neutralizing epitopes derived from the VP1 GH loop of type O FMDV. Viable chimeric viruses were produced with insertions located upstream of RGD +6, whilst chimeras with insertions downstream of this position were unable to be recovered. Although no *in vivo* studies were performed, neutralization assays identified a putative candidate with the potential to induce neutralizing antibodies against these two serotypes. In contrast to these studies, we have generated recombinant FMDV by insertion of exogenous tags (HA and FLAG) into an intact VP1 GH loop downstream of RGD +8. These epitope tags bind mAbs with high affinity, facilitating purification protocols to be developed – a strategy not possible with wild-type sequences. The tag insertion site was selected based on specific criteria to maintain the structural integrity of the capsid and infectiousness of the virus, and to provide accessibility to the epitope tags (Acharya *et al.*, 1989; Curry *et al.*, 1996; Logan *et al.*, 1993; Parry *et al.*, 1990). In particular, the insertion site needed to preserve the capability of the virus to utilize integrin-mediated cell entry (Burman *et al.*, 2006; Dicara *et al.*, 2008) and preserve accessibility to major antigenic site 1, involved in virus neutralization (Lea *et al.*, 1994, 1995). To this end, we demonstrated that FMDV expressing either the HA tag or the FLAG tag could interact with the principal FMDV receptor, integrin αvβ6, and produce plaques of similar size and morphology to cattle-passaged field virus and parental non-tagged virus. In addition, tagged viruses were recognized by major antigenic site 1-specific antibody, supporting the preservation and accessibility of this important site on the GH loop involved in virus neutralization. The HA tag was genetically stable over five passages. However, a virus subpopulation with an alteration to the sequence encoding the FLAG tag was detected during virus propagation. This was not an unexpected result; there are similar reports by other investigators on the genetic stability of the FLAG tag and examples of how the insert can be stabilized (Baranowski *et al.*, 2001; Prentoe & Bukh, 2011).

The inserted tags were shown to bind anti-tag antibodies that could be used to facilitate virus purification. The current conventional FMD vaccines consist of chemically inactivated whole virus preparations produced in cell culture. Such crude preparations contain high concentrations of cell and media components and unwanted virus components unless subjected to lengthy concentration and purification using techniques such as ultrafiltration and chromatography (Rodriguez & Grubman, 2009). Improvements in vaccine-purification techniques has given rise to a negative marker vaccine approach based on identifying infected animals by the presence of antibodies against NSP (Uttenthal *et al.*, 2010). Highly concentrated and purified FMDV capsid has the advantage of allowing the production of higher-quality vaccines and facilitates storage of concentrated materials as emergency antigen banks (Doel & Pullen, 1990). However, the current manufacturing techniques are technically demanding, only undertaken by some of the vaccine manufacturers and usually targeted for specific markets. These procedures do not completely remove all extraneous proteins and even highly purified preparations can contain detectable amounts of NSP that can induce a serological response in vaccinated animals, especially after multiple vaccinations (Doel, 2003; Lubroth *et al.*, 1998; Mackay, 1998; Paton & Taylor, 2011). There is therefore a need for simple, cost-effective antigen-purification techniques to improve the supply and availability of high-purity, high-quality conventional vaccines and to apply to the next generation of molecular vaccines (Rodriguez & Grubman, 2009). The inclusion of epitope tags on the FMDV capsid allows access to versatile antigen-purification methods based on simple affinity chromatography. We demonstrated that neutralization of tagged FMDV with anti-HA and anti-FLAG tag antibodies was reversible and that the tagged viruses were readily purified using anti-tag-coupled beads (both HA and FLAG). This allowed for elution at neutral pH under non-denaturing conditions by peptide competition, thus maintaining the structural integrity and therefore infectivity of eluted FMDV. Presumably, the observed neutralization in the presence of anti-tag antibodies was caused by steric hindrance of protein motifs involved in integrin-mediated cell entry. However, other causes such as conformational changes to the GH loop upon antibody binding cannot be ruled out.

FMDV expressing the FLAG tag provides access to immunoaffinity chromatography and elution under non-denaturing conditions by taking advantage of calcium-dependent antibodies and elution with chelating agents or competition elution (Einhauer & Jungbauer, 2001). Access to these routine antigen-purification techniques provides an option to simplify FMDV antigen concentration and purification for both researchers and vaccine manufacturers. Further studies are currently in progress to improve the efficiency of purification and to investigate the efficacy of purified, tagged FMDV capsids as a vaccine candidate.

The ability to produce tagged FMDV capsid provides a valuable tool for studying the FMDV life cycle, including investigation of virus entry pathways, uncoating and capsid synthesis. In addition, tagged FMDV capsids facilitate the development of FMDV vaccine conjugates and immune targeting. A number of approaches have been described for human vaccine conjugates, e.g. to target cells of the immune system like dendritic cells and B-cells, or triggering Toll-like receptor signalling to regulate T-cell immunity (Ueno *et al.*, 2011; van Duin *et al.*, 2006). Current FMD vaccines induce high levels of neutralizing antibody; however, the short duration of protection that they elicit is a major disadvantage that hinders control policies (Cox & Barnett, 2009). There is evidence to suggest that the current FMD vaccines do not stimulate the strong and reproducible T-cell responses required for the development of long-lasting neutralizing antibody responses (Carr *et al.*, 2008; Ochsenbein *et al.*, 2000; Robinson *et al.*, 2008). Robinson *et al.* (2011) targeted UV-inactivated antibody-complexed FMDV to dendritic cells via CD32. This led to a significant increase of the *in vitro* T-cell restimulation response, suggesting that FMD vaccines may be more effective when targeted to dendritic cells (Robinson *et al.*, 2011). Studies utilizing antibodies directed against the dendritic-cell receptor DEC205 have demonstrated that dendritic-cell targeting results in potent induction of antigen-specific CD4^+^ and CD8^+^ T-cell responses and enhanced resistance to virus infection at mucosal sites (Bonifaz *et al.*, 2004). A similar approach has been used to specifically induce cytokines critical for resistance to infection and tumours (Soares *et al.*, 2007). There is also evidence in the literature that antigen is more stable in the form of immune complexes, for example the enhanced stability of immune-complexed human immunodeficiency virus (Griffith *et al.*, 1995). Our future experiments will focus on the degradative characteristics, thermostability and the affect on antigen processing and presentation of conjugated FMDV.

In conclusion, we have epitope-tagged the VP1 SP of FMDV in a region that maintains the structural integrity and virus life cycle. The tags are accessible to antibodies and provide a unique tool to investigate infections *in vivo* and to characterize cellular events from cell entry to the release of infectious virions. Moreover, tagged FMDV can be purified to a high level and offers an alternative method of purification for conventional and next-generation empty-capsid vaccines.

## Methods

### 

#### Construction of epitope-tagged viruses.

Infectious tagged FMDV O1K/O UKG35 and tagged FMDV O1K/O1Manisa (O1M) chimeric clones were constructed using reverse genetics. Briefly, cDNA encoding the VP2, VP3, VP1 and 2A proteins was removed from a derivative of the pT7S3 O1K infectious clone, termed pT7SBmuts, leaving cDNA encoding the Lpro, VP4, 2B, 2C, 3A, 3B, 3C and 3D proteins (Bøtner *et al.*, 2011). The removed cDNA was replaced with the corresponding O UKG35 or O1M cDNA from pGEM9zf subclones encoding HA (YPYDVPDYA) or FLAG (DYKDDDDK) epitope tags in the GH loop of VP1 (Ellard *et al.*, 1999; Zibert *et al.*, 1990) (UKG/35/2001; GenBank accession no. AJ539141). DNA encoding the respective peptide tag was inserted into the subclones by performing three consecutive rounds of PCR amplification using a QuikChange Lightning Mutagenesis kit (Agilent Technologies) according to the manufacturer’s instructions. The primers used for insertion of FLAG and HA tags are listed in Table S1 (available in JGV Online). RNA was then transcribed from the full-length ‘tagged’ infectious clones using a MEGAscript T7 kit (Invitrogen). The infectious RNA was electroporated into BHK-21 cells using a Bio-Rad Gene PulsarTM (two pulses at 0.75 kV and 25 μF). After 24 h, the cells were freeze–thawed in their growth medium and clarified by centrifugation at 2095 ***g*** for 10 min, the supernatant of which contained the initial virus stock [termed ‘passage 0’ (P0)]. A goat epithelium cell line was subsequently used to passage the tagged viruses (P1) (Brehm *et al.*, 2009). Cells were infected for 24 h between passages.

#### Genome amplification and sequencing.

Total RNA was extracted using TRIzol reagent (Invitrogen) and the respective region of the viral RNA genome was reverse-transcribed and amplified by PCR using a One-Step RT-PCR kit (Qiagen). Sequencing reactions were then performed using an aliquot of the purified PCR product and a BIG Dye Terminator v. 3.1 cycle sequencing kit (Applied Biosystems).

#### Western blot analysis.

For Western blots, proteins were separated by SDS-PAGE (12 % acrylamide) and then transferred to nitrocellulose membranes (Hybond-C Extra; Amersham Biosciences). Membranes were blocked with dried skimmed milk in PBS containing 0.1 % Tween 20. Primary mAbs used were D9 [mouse anti-FMDV major antigenic site 1 (McCahon *et al.*, 1989)], 2C2 [mouse anti-FMDV 3A (De Diego *et al.*, 1997)], mouse anti-HA tag [HA-7 (Sigma-Aldrich)], rabbit anti-HA tag [715500 (Invitrogen)], goat anti-HA tag (QED Bioscience) and mouse anti-FLAG tag [M2 (Agilent Technologies)]. Bound primary antibodies were detected by HRP-conjugated anti-mouse or anti-rabbit antibodies (Bio-Rad).

#### Immunofluorescence microscopy.

Goat epithelium cells (ZZ-R127) (Brehm *et al.*, 2009) cultured on glass coverslips were incubated with P1 HA- or FLAG-tagged FMDV O1K/O UKG35; non-infected cells were included as a control. After 4 h, cells were fixed, permeabilized, washed and blocked (0.5 % BSA in PBS). HA and FLAG tags were labelled with mouse anti-HA (HA-7; Sigma-Aldrich) and mouse anti-FLAG M2 antibodies (M2; Agilent Technologies). FMDV non-structural protein 3A was labelled using mAb 2C2 (De Diego *et al.*, 1997) and FMDV capsid was labelled using mAb BF8 (Juleff *et al.*, 2008). Goat anti-mouse Molecular Probes Alexa Fluor-conjugated secondary antibodies (Invitrogen) were used and nuclei were stained with DAPI (Sigma-Aldrich). All data were collected sequentially using a Leica SP2 laser scanning confocal microscope.

#### Plaque assay.

Confluent monolayers of goat epithelium cells were infected with serial dilutions of FMDV O1K/O UKG35 virus stocks, overlaid with indubiose and incubated for 24–48 h at 37 °C. The cells were then fixed and stained (4 % formaldehyde in PBS containing methylene blue) before removal of the overlay (Jackson *et al.*, 2000). FMD lesion vesicular fluid from a cow infected with FMDV O/UKG 34/2001 was included as a positive control.

#### Integrin-blocking assay.

Twofold-dilution series of recombinant integrin αvβ6 in PBS were made (from columns 2–11 of 96-well cell culture plates) from a starting concentration of 10 µg ml^−1^ (kindly provided by N. Ferris and produced by N. Abrescia, University of Oxford, UK) (Burman *et al.*, 2006; Ferris *et al.*, 2005). A fixed 1/10 dilution of HA- or FLAG-tagged FMDV O1K/O UKG35 tissue-culture supernatant was then added in six replicates for each virus. After incubation at room temperature for 30 min, the suspensions were added to confluent goat epithelium cells in 96-well cell culture plates. A virus control (column 1) and cell control (column 12), with the highest concentration of integrin, were included. The plates were incubated at 37 °C for 24 h, fixed in 4 % paraformaldehyde and stained with a solution of naphthalene black in citric acid (Sigma-Aldrich).

#### Antibody-blocking assay.

Antibody-blocking assays were carried out using mAbs recognizing the HA and FLAG tags [HA-7 (Sigma-Aldrich) and M2 (Agilent Technologies), respectively]. Blocking assays were performed as described for plaque assays, with the exception that diluted viruses were incubated with the anti-FLAG or anti-HA antibody, or both, for 15 min at room temperature prior to cell infection.

#### FMDV antigen-detection ELISA.

An anti-FMDV sandwich ELISA was used to measure the interaction of FMD O1K/O UKG35 non-tagged or FLAG- and HA-tagged viruses with mouse mAb D9, an antibody directed against major antigenic site 1 (McCahon *et al.*, 1989). Ninety-six-well Maxisorb Nunc immunoplates (Sigma-Aldrich) were coated overnight at 4 °C with a solution of rabbit anti-O1 Manisa hyperimmune antiserum (1 : 1000) in 0.1 M carbonate–bicarbonate buffer. Coated plates were washed and incubated with triplicate twofold dilutions series of 1×10^6^ p.f.u. ml^−1^ of each virus at a starting dilution of 1/4 to 1/128. Plates were washed and blocked with sodium casein (Sigma-Aldrich), and FMDV antigen was detected using purified D9 antibody at 0.75 µg ml^−1^ followed by HRP-conjugated rabbit anti-mouse IgG (DakoCytomation). Plates were developed with TMB Stabilized Substrate (Promega) and the reaction was stopped with the addition of 0.2 M sulphuric acid. *A*_450_ values were read on a Synergy 2 Multi Detection microplate reader (Biotek).

#### Immunoaffinity purification.

For each purification, a T175 flask containing a confluent monolayer of goat epithelium cells was infected with epitope-tagged FMDV O1K/O UKG35 (P1 stock) at an m.o.i. of 0.1 for 20 h. Upon observation of CPE, the cells were freeze–thawed in their growth medium and clarified by centrifugation at 2100 ***g***. Two hundred microlitres of a 50 % suspension of equilibrated anti-FLAG (M2) or anti-HA (HA-7) agarose bead resin (Sigma-Aldrich) was added and incubated with mixing by rotation for 1 h at room temperature. Beads were washed three times, each with 50 ml PBS and precipitation by centrifugation at 250 ***g***. Tagged viruses were eluted three times by competition with either HA–peptide or FLAG–peptide (both from Sigma-Aldrich) at a concentration of 100 µg ml^−1^. Samples of eluted virus were analysed by SDS-PAGE and silver staining using a commercial silver-staining kit (Pierce, Thermo Scientific).

#### Llama single-domain antibody ELISA.

Llama single-domain antibody fragments (VHH domains) against FMD viral 146S particles (VHH-M170F) were provided by the Central Veterinary Institute of Wageningen UR, AB Lelystad, the Netherlands (Harmsen *et al.*, 2007). The wells of a 96-well microtitre plate were incubated overnight with VHH-M170F in carbonate/bicarbonate buffer (50 mM, pH 9.6; Sigma-Aldrich) at a concentration of 0.5 mg l^−1^. Test samples diluted serially in VHH buffer (1 % skimmed milk; 0.05 % Tween; 0.5 M NaCl; 2.7 mM KCl; 2.8 mM KH_2_PO_4_; 8.1 mM Na_2_HPO_4_, pH 7.4) were added to the wells and incubated for 1 h at 37 °C. Next, the biotinylated VHH-M170F, diluted in VHH buffer at a concentration of 0.1 mg l^−1^, was added and incubated for 1 h at 37 °C. Plates were washed with PBS–Tween (0.05 % Tween 20) after coating, sample application and incubation with biotinylated VHH. Bound biotinylated VHH-M170F was visualized by incubation with streptavidin–HRP (Dako) for 1 h at 37 °C, followed by addition of *o*-phenylenediamine dihydrochloride (Sigma-Aldrich) for 15 min at room temperature. The reaction was stopped by addition of 1.25 M sulphuric acid and *A*_490_ was quantified using a Dynex microplate reader (Dynex Technologies).
